# A Novel Tactile Sensor with Electromagnetic Induction and Its Application on Stick-Slip Interaction Detection

**DOI:** 10.3390/s16040430

**Published:** 2016-03-24

**Authors:** Yanjie Liu, Haijun Han, Tao Liu, Jingang Yi, Qingguo Li, Yoshio Inoue

**Affiliations:** 1 State Key Laboratory of Robotics and System, Department of Mechatronics Engineering, Harbin Institute of Technology, Harbin 150001, China; yjliu@hit.edu.cn; 2 State Key Laboratory of Fluid Power and Mechatronic Systems, College of Mechanical Engineering, Zhejiang University, Hangzhou 310058, China; 3 Department of Mechanical and Aerospace Engineering, Rutgers University, Piscataway, NJ 08854, USA; jgyi@rutgers.edu; 4 Department of Mechanical and Materials Engineering, Queen’s University, Kingston, ON K7L3N6, Canada; ql3@queensu.ca; 5 Research Institute, Kochi University of Technology, Kochi 782-0003, Japan; inoue.yoshio@kochi-tech.ac.jp

**Keywords:** tactile sensor, electromagnetic induction, stick-slip detection, end effector, wafer transfer robot

## Abstract

Real-time detection of contact states, such as stick-slip interaction between a robot and an object on its end effector, is crucial for the robot to grasp and manipulate the object steadily. This paper presents a novel tactile sensor based on electromagnetic induction and its application on stick-slip interaction. An equivalent cantilever-beam model of the tactile sensor was built and capable of constructing the relationship between the sensor output and the friction applied on the sensor. With the tactile sensor, a new method to detect stick-slip interaction on the contact surface between the object and the sensor is proposed based on the characteristics of friction change. Furthermore, a prototype was developed for a typical application, stable wafer transferring on a wafer transfer robot, by considering the spatial magnetic field distribution and the sensor size according to the requirements of wafer transfer. The experimental results validate the sensing mechanism of the tactile sensor and verify its feasibility of detecting stick-slip on the contact surface between the wafer and the sensor. The sensing mechanism also provides a new approach to detect the contact state on the soft-rigid surface in other robot-environment interaction systems.

## 1. Introduction

Robot-environment interaction is common in robotics applications [1], such as grasping and manipulating objects [2], bipedal walking [3], *etc*. A tactile sensors is a device or system capable of measuring the contact parameters between the sensor and an object [4], such as contact force and slippage, and provide an important approach for robots to sense the environment, like the sense of touch in humans. In recent years, various tactile sensors have been developed for robotic applications [4,5,6], such as humanoid dexterous hands [6,7], minimally-invasive surgery robots [8,9], rehabilitation robots [10], *etc*. Among them, slip sensing is an important approach to recognize incipient (or micro) and total (or macro) manipulation of an object relative to the sensor [11], which is crucial for stable grasping and manipulating of the object [12].

In general, tactile sensors with slip perception are mainly based on three principles. The first one is to estimate the friction coefficient or friction angle indirectly from normal and tangential forces, which are often measured by multi-dimensional force sensors [4,5]. However, it is not an accurate indirect detection, and most of multi-dimensional force sensors are not able to capture slipping instantly, especially incipient slip [13], due to poor dynamic properties. Their miniaturization is also a problem. The second one is to analyze the changes in the contact shape with the object and changes in the pressure distribution based on array/matricial sensors or skin sensors [14]. However, it is not useful if the object is not rigid, and the array sensors are always limited by low spatial resolution and a high cost. The third technique is to detect slip-induced micro-vibration by using dedicated sensors with accelerometers, acoustic detectors, or piezoelectric materials [15]. This approach is a direct detection and always has good dynamic properties. However, most of them are developed for specific applications and with some shortcomings due to the properties of their sensing materials. The readers can refer to a recent review [11] about slip detection for more details.

In this paper, a novel tactile sensor with electromagnetic induction is proposed to detect stick-slip interaction. Here, “stick” means that the sensor and the object remain relatively stationary, and “slip” means the object slides on the sensor. Since the maximum static friction is usually greater than the sliding friction for most of friction pairs, there is a transition state from “stick” to “slip”, defined as “stick-slip” in this paper. To some extent, stick-slip can be considered as a short-term process that sliding appears in a local contact region and then suddenly extends to the whole contact region [16,17,18]. Thus, stick-slip detection is crucial to slip perception, especially for incipient slip. The electromagnetic induction principle is widely used for sensors, but mostly with displacement [19] or velocity measurement [20]. Wattansarn *et al*. developed a three-dimensional tactile sensor with two layers of induction coils, one layer for signal excitation and the other for reception [21]. However, its force detection precision is low since its force model just comes from the experimental calibration.

**Figure 1:**
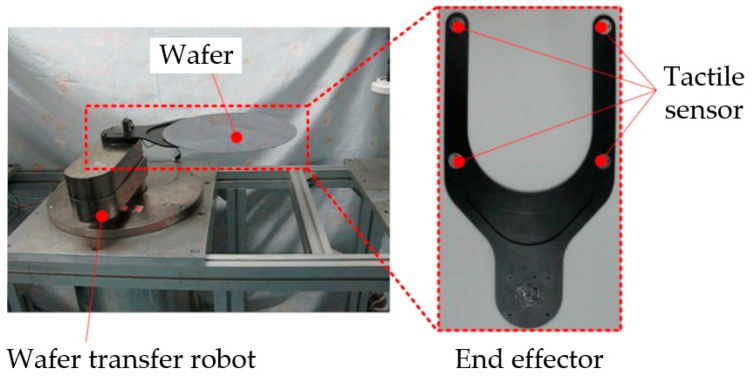
A typical wafer transfer robot with a wafer and a schematic of tactile sensor application.

The proposed tactile sensor is inspired by a novel electromagnetic induction-based velocity sensor we proposed in [22]. However, the velocity is unable to be used directly for detecting stick-slip interaction. In this paper, an equivalent cantilever-beam model is built to construct the relationship between the output of tactile sensor and the friction on the contact surface. Furthermore, a new method based on the tactile sensor is proposed to detect stick-slip interaction. Then, we developed a prototype for stick-slip interaction testing on a wafer transfer robot (Figure 1). Wafer transfer robots are widely used in the fabrication of integrated circuit to transfer wafers precisely, fast, and steadily between different wafer processing stations [23]. Stick-slip detection between the wafer and the end effector of the robot provides crucial information for accessing the quality of trajectory planning of the robot and improving the performance of robotic control [24,25,26]. Due to space constraints (only 10 mm in height between layers in a standard 300-mm wafer cassette) and avoiding a large additional load, a compact and light tactile sensor is desirable for wafer transfer, but few of the current tactile sensors meet this requirement. Thus, we developed this prototype by considering spatial magnetic field distribution and sensor size for wafer transfer, and conducted the experiments for sensor calibration and model validation.

The rest of the paper is structured as follows. The structure and mechanism of tactile sensor is described in the second section, as well as the method to detect stick-slip. In the third section, we present the design of tactile sensor in details. The experiment and result analysis are presented in the fourth section. The fifth and sixth sections contain the discussion and conclusion, respectively.

## 2. Materials and Methods

### 2.1. Structure and Mechanism of Tactile Sensor

**Figure 2:**
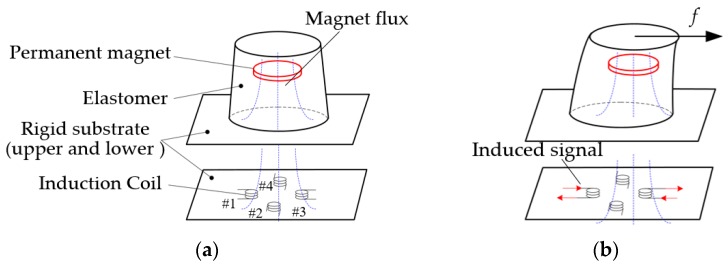
Schematic diagram of the tactile sensor. (**a**) Configuration of the tactile sensor with a permanent magnet, an elastomer, and four small chip inductors (#1~#4); (**b**) The induced voltage is generated in the inductor under the deformation of the elastomer with the magnet.

Figure 2 illustrates the structure of tactile sensor. It contains a cylindrical permanent magnet, an elastomer, a rigid substrate, and four small chip inductors (#1~#4). The magnet is embedded into the elastomer which is mounted on the upper surface of rigid substrate. The four inductors are bonded with circular symmetrical distribution on the lower surface of rigid substrate and under the elastomer. When a tangential force, such as the friction, is applied on the sensor (Figure 2b), the elastomer will deform and drive the magnet moving over the coils to cut the magnetic induction line. At the same time, the induced voltage will be generated in the inductors due to the Faraday’s law of electromagnetic induction.

**Figure 3:**
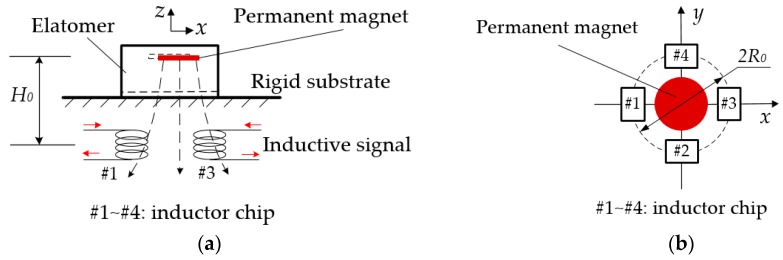
Mechanism of the tactile sensor. (**a**) Cross-sectional view; and (**b**) top view.

Let *H*_0_ and *R*_0_ denote the height of the magnet *versus* the plane of inductive chip array and the radius of the distribution circle of the array, respectively, and set the coordinate system as shown in Figure 3.

The relation between the amplitude of induced voltage (*U_out_*) and the magnet flux (Φ) can be obtained by Faraday’s law as follows [27]:

(1) $$U_{out}=-N\frac{d\Phi }{dt}=-N\frac{d(\int_{S}B_{⊥}(s)ds)}{dt}$$

where *N*, *S*, and *B*_⏊_ are the number of coils in a chip inductor, the surface area of a coil, and the vertical component of magnetic flux density through the coil plane, respectively.

When *S* is small enough (≤0.5 mm^2^), it can be regarded as a constant and placed outside the integral sign as follows:

(2) $$U_{out}\approx -NS\frac{dB_{⊥}}{dt}$$

Considering the magnet moving in the radial direction of the distribution circle, Equation (2) can be rewritten as follows:

(3) $$U_{out}\approx -NS\frac{dB_{⊥}}{dt}\approx -NS\frac{dB_{z}}{dr}v_{r}$$

where *v_r_* = *dr*/*dt* denotes the radial component of the magnet velocity.

When the magnet moves with a very small displacement, *dB_z_*/*dr* can be regarded as a constant, and then:

(4) $$U_{out}=-A_{C}v_{r}$$

where *A_C_* is a constant related to sensor structure and can be calibrated by experiments.

When four identical inductors are deployed differentially (#1 and #3, #2 and #4, see Figure 3b), there are relationships between two couples of outputs and velocity components of the magnets as follows:

(5) $$\left\{ \begin{array}{l} v_{x}=K_{Cx}(U_{1}-U_{3})=K_{Cx}U_{13}\\ v_{y}=K_{Cy}(U_{2}-U_{4})=K_{Cy}U_{24} \end{array}\right.$$

where *K_Ci_* is a constant related to *A_C_*, *i* = *x*, *y* indicates that along *x*-axis or *y*-axis.

**Figure 4:**
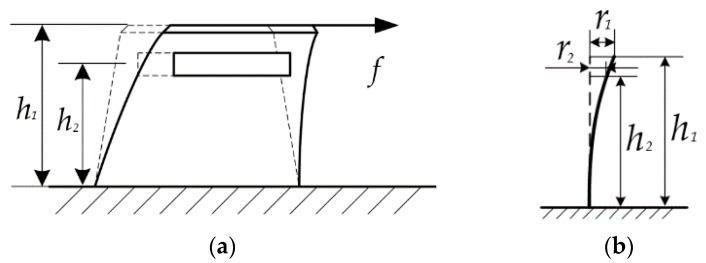
The model of the tactile sensor. (**a**) The elastomer is deformed under the frictional force; and (**b**) the equivalent cantilever-beam model for the tactile sensor.

Let *h*_1_ and *h*_2_ denote the height of the elastomer and that of the magnet (Figure 4a), respectively. Due to the elastic characteristic of elastomer, the whole elastomer can be considered as a cantilever beam as shown in Figure 4b. Under the frictional force *f*, the deflection of elastomer in tangential direction is set as *r*_1_, and the corresponding displacement of magnet is *r*_2_.

In terms of material mechanics [28], we can obtain the following equation:

(6) $$r_{2}=\frac{fh_{2}^{2}}{6EI}\left(3h_{1}-h_{2}\right)$$

where *E* and *I* are the equivalent Young’s modulus and the equivalent moment of inertia, respectively. Further, the following equation can be obtained from Equation (6):

(7) $$f=\frac{6EI}{h_{2}^{2}\left(3h_{1}-h_{2}\right)}r_{2}=\frac{6EI}{h_{2}^{2}\left(3h_{1}-h_{2}\right)}\int_{t}v_{r}dt=k\int_{t}v_{r}dt$$

where *v_r_* = *dr*_2_/*dt* is the same as that in Equation (3), that is, the velocity of the magnet in a tangential direction, and *k* is a constant related to the structure and material of the sensor.

With Equations (4)–(7), it indicates that the frictional force *f* can be obtained from the outputs of the sensor. Therefore, it provides an approach to use the proposed tactile sensor to measure the friction force, which can be further used to detect the contact state on contact surface.

### 2.2. Stick-Slip Detection Method Based on the Tactile Sensor

For determining whether the stick-slip appears or not, it is necessary to capture the change of friction by an effective method due to the characteristics of friction in the “stick”, “stick-slip”, and “slip” states. Differential function is a common method to capture the change of the original signal. Thus, we define the derivation of friction as a judgment function *J* (*t*):

(8) J(t)=df(t)/dt

In practical application, *J* (*t*) can be calculated or measured in real time and compared with a threshold to determine whether the stick-slip appears or not. Substituting Equation (7) into Equation (8), we can obtain:

(9) $$J\left(t\right)=k\cdot v_{r}^{}$$

Substituting Equation (5) into Equation (9), we can obtain that:

(10) $$\left\{ \begin{array}{l} J_{x}\left(t\right)=K_{Cx}kU_{13}\left(t\right)\\ J_{y}\left(t\right)=K_{Cy}kU_{24}\left(t\right) \end{array}\right.$$

Further, as *K_Ci_k* (*i* = *x*, *y*) is a constant, the judgment function *J* (*t*) can be obtained from the output of tactile sensor directly as follows:

(11) $$\left\{ \begin{array}{l} J_{x}\left(t\right)=U_{13}\left(t\right)\\ J_{y}\left(t\right)=U_{24}\left(t\right) \end{array}\right.$$

which indicates that the outputs of tactile sensor can be used directly to detect the stick-slip.

## 3. Tactile Sensor Design

As the space of each layer in a standard 300-mm wafer cassette is only 10 mm in height, it requires that the height of the tactile sensor should be as low as possible so that the end effector can get into and out of the wafer cassette easily. Meanwhile, the mass of the tactile sensor should be as small as possible in order to reduce its impact on the vibration of the end effector when the robot is moving. Therefore, the tactile sensor should be as compact and light as possible. This section demonstrates the design of tactile sensor in detail.

### 3.1. Magnetic Field Distribution of Cylindrical Permanent Magnet

According to the principle of tactile sensor, it is essential that *dB_z_*/*dr* should be regarded as a constant. Hence, it is necessary to obtain the spatial magnetic field distribution formulas of cylindrical permanent magnet firstly and explore an available range of *dB_z_*/*dr* for the tactile sensor.

**Figure 5:**
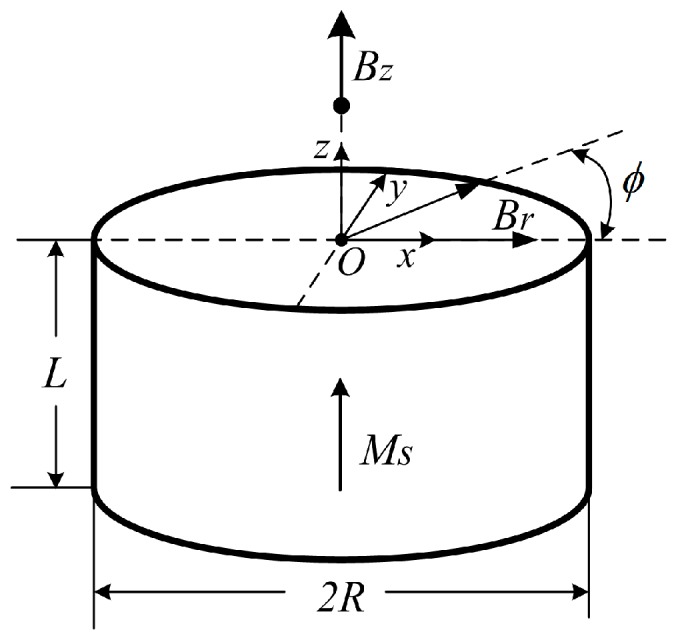
Sketch of an axially-polarized cylindrical permanent magnet.

For magnetic field analysis of permanent magnets, there are two models, the current model and the charge model, both of which are used to reduce a permanent magnet to an equivalent source term and have the same results [29]. Here, the current model is used. In this model, the magnet is reduced to a distribution of equivalent current which is then input into the magneto-static field equations as a source term, and the field is obtained using standard methods for steady currents. The model of an axially-polarized cylindrical permanent magnet is shown in Figure 5.

Let a *O*-*xyz* coordinate system be built on the top of the magnet and *R*, *L* denote the radius and height of the magnet, respectively. Assume that the magnet has a second quadrant demagnetization curve, that is, $B=\mu_{0}(H+M_{s}\hat{z})$, where $\mu_{0}$, H, $M_{s}$, $\hat{z}$ are the permeability, the magnetic field strength, the saturation magnetization, and the *z*-axis unit vector, respectively. Based on the current model, the radial and axial components of magnetic flux density **B** (*B_r_* and *B_z_*) can be derived as follows:

(12) $$\left\{ \begin{array}{c} B_{r}(r,\phi ,z)=\frac{\mu_{0}M_{s}}{4\pi }\sum_{k=1}^{2}{\left(-1\right)}^{k}\int_{0}^{2\pi }\cos(\phi -\phi \prime )g(r,\phi ,z;R,\phi \prime ,z_{k})Rd\phi \\ B_{z}(x)=\frac{\mu_{0}M_{s}}{4\pi }{\sum_{j=1}^{2}{\left(-1\right)}^{\left(j+1\right)}\int_{z_{1}}^{z_{2}}\int_{0}^{2\pi }\frac{r\cos(\phi -\phi \prime )-R_{c}(j)}{{\left|x-x\prime \right|}^{3}}|}_{r\prime =R_{c}(j)}R_{c}(j)d\phi dz \end{array}\right.$$

where *r*, ϕ, *x* are the radial magnitude, rotation angle (with *x*-axis), and *x*-axis component of the vector from the observation point to *O*, respectively. Correspondingly, ϕ′ and **x**’ are those from the reference point to *O* (usually 0 or **0**). Here, $g(r,\phi ,z;R,\phi \prime ,z_{k})={\left[r^{2}+R^{2}-2rR\cos(\phi -\phi \prime )+{\left(z-z\prime \right)}^{2}\right]}^{-1/2}$, where *z* and *z*′ are the *z*-axis position of the observation point and the reference point, respectively. *z*_1_ and *z*_2_ are the axial position of magnet bottom and top surface, respectively. For more details about the derivation of Equation (12), please refer to [29].

From Equation (12), it can be inferred that the magnetic flux density at any point outside of an axially-polarized cylindrical magnet is only the function of its position. Furthermore, Equation (12) can be dealt with numerical method to calculate the magnetic field distribution of the cylindrical permanent magnet with arbitrary size.

### 3.2. Size Determination for the Magnet and the Chip-Inductor Array

**Figure 6:**
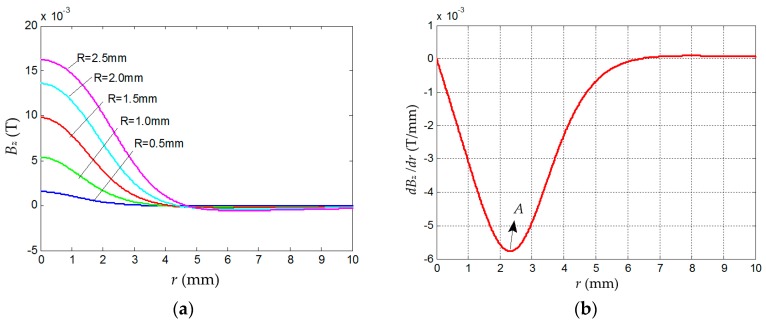
Simulation results. (**a**) *B_z_* distribution simulation of NdFeB magnet with different radius (*R*); and (**b**) *dB_z_*/*dr* distribution of NdFeB magnet (*R* = 2.5 mm and *L* = 0.5 mm) *versus* *r* in the radial direction.

Considering the layer space of the wafer cassette and the general thickness of an end effector, the height of the tactile sensor should be less than 5 mm. Taking the elastic deformation of the elastomer and the volume restriction of sensor into account, let *H*_0_ = 3.0 mm (Figure 3), *L* = 0.5 mm and *R* ≤ 2.5 mm (Figure 5). To reduce costs, we choose a type of commercial cylindrical permanent magnet (Sangyo Supply Co., Tokyo, Japan), whose material is NdFeB, with a compact structure and powerful magnetism. There are five sizes of magnet meeting the requirements, that is, *R* = 0.5 mm, 1.0 mm, 1.5 mm, 2.0 mm, and 2.5 mm. To determine the right size, the magnetic field distribution of the magnets of different size are calculated with the numerical method by using MATLAB (2010R, The MathWorks Co., Natick, MA, USA). The simulation result is illustrated in Figure 6a, which shows that both the amplitude and linear region of *B_z_* in the radial direction increase with *R* increasing. Therefore, the magnet with *R* = 2.5 mm and *L* = 0.5 mm is selected here.

According to the mechanism of tactile sensor, to lower measurement error, *dB_z_*/*dr*, should be approximately a constant. Meanwhile, the larger *dB_z_*/*dr* is, the larger the output of the sensor is. To find a qualified range, *dB_z_*/*dr* of the selected-size magnet *versus* its radius (*r*) is calculated by the numerical method and the result is shown in Figure 6b, which indicates that there is an extreme point *A* at *r* = 2.33 mm, where |*dB_z_*/*dr*|_max_ = 5.76 × 10^−3^ T/mm. Hence, *R*_0_ (Figure 3b) is set to 2.33 mm. Assuming that the linear range is determined by *Δd*/*d* = 0.5%, where *d* is equal to the value of *dB_z_*/*dr* at point *A*, it can be obtained that the corresponding radial range is |*Δr*| = 0.25 mm, which means if the deflection of the elastomer (*r*_1_ in Figure 4) is less than 0.25 mm, the linearity of the sensor output is less than 0.5%.

### 3.3. Prototype Development of Tactile Sensor

**Figure 7:**
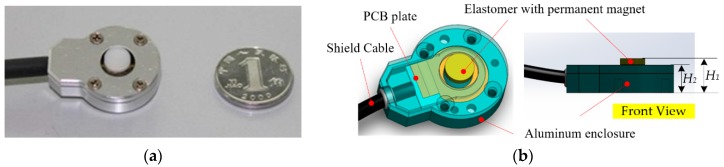
The prototype of tactile sensor. (**a**) The prototype and a coin of 1 Jiao RMB (Chinese currency) which is 19 mm in diameter; and (**b**) the structure of the prototype.

Considering all above requirements, a prototype of tactile sensor is designed and fabricated as shown in Figure 7. The prototype contains a rubber elastomer, a cylindrical NdFeB magnet, a printed-circuit-board (PCB) plate for supporting the elastomer and signal transmission, a thin shield cable for signal transmission, and two enclosures made of aluminum alloy for covering the whole sensor and shielding the external high-frequency signal. Due to the compact structure and light materials, the whole sensor is 8.0 mm in height (*H*_1_ = 8.0 mm, *H*_2_ = 6.0 mm in Figure 7), 20 mm in diameter and around 26 g in weight.

## 4. Experiments

**Figure 8:**
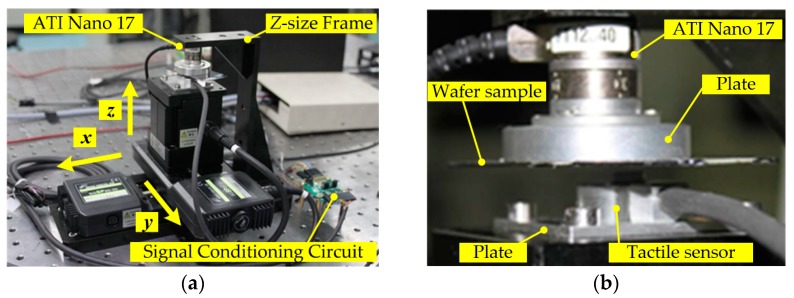
Calibration setup for tactile sensor. (**a**) The setup with the 3D motion stage and the ATI Nano 17; and (**b**) the enlarged view of the contact between a wafer sample and the prototype.

To investigate the performance of proposed tactile sensor and confirm the aforementioned models, experiments should be conducted. Thus, an experimental setup is established as shown in Figure 8.

The setup mainly consists of the tactile sensor, a three-dimensional (3D) micro-motion platform and a six-axis force/torque (F/T) sensor. The 3D micro-motion platform (SGSP20-35XY and SGSP60-5ZF, Sigma Koki Co., Ltd., Tokyo, Japan) is capable of moving with a standard trapezoidal speed-time curve, and has a repeatability of 5 µm along the *x*-axis or the *y*-axis and 10 µm along the *z*-axis, as well as a maximum speed of 25 mm/s along the *x*-axis or the *y*-axis and 2 mm/s along the *z*-axis. It is installed on an isolation vibration platform. The tactile sensor is mounted on the top of 3D motion platform through a connection plate and four bolts. The F/T sensor (Nano 17 SI-12-0.12, ATI Industrial Automation, Apex, NC, USA) has a resolution of 38 mN along the *x*-axis or the *y*-axis and 53 mN along the *z*-axis, with a resonant frequency of 7 kHz along each axis. It is fixed on a “Z”-size aluminum alloy frame which is mounted on the isolation vibration platform. A piece of a wafer sample is glued on a connection plate, which is connected with the F/T sensor by bolts, in order to imitate the condition for the real application of wafer transfer.

The output signal of tactile sensor is pre-processed by a customized signal conditioning circuit mainly including two precision instrument amplifiers (AD 8221, Analog Devices, Inc., Norwood, MA, USA) and a Butterworth low-pass filter with a cutoff frequency of 6 kHz. The total gain is about 5000. Then, the signal is input into a data acquisition card (NI PCIe-6323, National Instruments Co., LTD., Austin, TX, USA) with 32 analog inputs (16-bit A/D, ±10 V), and 250 kS/s sampling rate (1-channel). The outputs of Nano 17 are also connected with the PCIe-6323. Furthermore, NI LabVIEW (2010, National Instruments Co., LTD., Austin, TX, USA) is used to build the program integrating motion control and synchronous measurement.

### 4.1. Tactile Sensor Calibration and Model Validation

For utilizing the tactile sensor to detect stick-slip interaction on a wafer transfer robot, the sensor calibration and model validation should be conducted firstly. Due to the symmetric structure of the tactile sensor, the process of calibration in the *x*- and *y*- direction are similar. Thus, here we only describe the case in the *x*-direction. Firstly, we make the tactile sensor move up to contact the wafer sample. To determine whether the contact occurs, a threshold *F_z_*_-threshold_ is used; that is, the contact occurs when *F_z_* ≥ *F_z_*_-threshold_. To ensure a good contact, *F_z_*_-threshold_ is set around 1.0 N. Then, let the tactile sensor move back and forth under the drive of the motion platform with a standard trapezoidal speed curve in the x-direction and its maximum absolute speed (*V*_max_) as a constant for one case, while simultaneously recording the speed (*V_x_*) of the tactile sensor and the output of the tactile sensor after pre-processing (*U_x_*, the input to NI PCIe-6323) with respect to time. Meanwhile, the displacement of the tactile sensor (*s*) is required to remain in a small range in order to keep a good, relatively stationary position between the top of tactile sensor and the wafer, setting |*s*| ≤ 1 mm, and then *V_x_* can be considered as the relative velocity between the top of the tactile sensor and its bottom.

**Figure 9:**
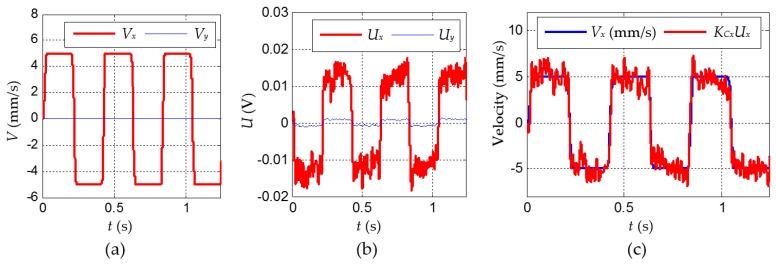
The results under the case that *V*_max_ = 5 mm/s. (**a**) The velocity of tactile sensor over the ground; (**b**) the output of the tactile sensor *U*; and (**c**) the result of *V_x_* and *K_Cx_U_x_*.

Figure 9 illustrates the experimental results in one typical case; that is, the tactile sensor moves back and forth under *V*_max_ = 5 mm/s (Figure 9a). *U_x_* is sampled by 5 kHz and then filtered by a digital Butterworth low-pass filter with the cutoff frequency of 100 Hz for reducing the high-frequency noise. The result (Figure 9b) shows that the output of the tactile sensor has a similar trajectory as that of the velocity. Here, the state when *V_x_* = *V*_max-*x*_ can be considered as the steady output of the tactile sensor, namely |*U_x_*|_max_ ≈ 8 × 10^−3^ V. Let *K_Cx_* = − *V*_max_/|*U*_x_|_max_, where the minus sign means the opposite direction, and we obtain *K_Cx_* ≈ 609.8 mm/(sV). Figure 9c shows *V_x_* and *K_Cx_U_x_* are in a combined figure. This indicates that the output of tactile sensor matches well with the speed curve, which implies that the tactile sensor is capable of measuring the velocity.

**Figure 10:**
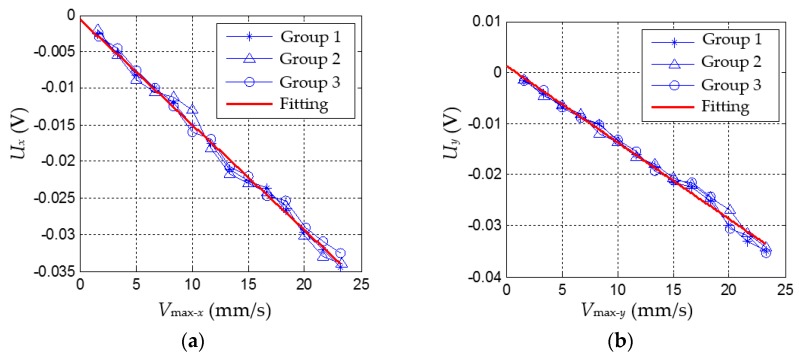
The calibration result of tactile sensor. (**a**) The calibration result of tactile sensor in the *x*-direction; and (**b**) the calibration result of tactile sensor in the *y*-direction.

Furthermore, let *V_max_* increase step by step from 1 mm/s to 24 mm/s and for each case do the same experiment as that under *V_max_* = 5 mm/s. Then, the corresponding |*U_x_*|_max_ can be obtained for each *V_max_*. Figure 10 shows the results of tactile sensor under different *V_max_* in the *x*- and *y*- direction. For each direction, we conducted three groups of experiments with *V_max_* from 1 mm/s to 24 mm/s.

By using the polynomial fitting method of MATLAB, the relationship between *V_i_* and *U_i_* (*i* = *x*, *y*) can be obtained as follows:

(13) $$\left\{ \begin{array}{l} U_{x}=V_{x}/K_{Cx}+b_{x}\\ U_{y}=V_{y}/K_{Cy}+b_{y} \end{array}\right.$$

where *K_Ci_* and *b_i_* (*i* = *x*, *y*) are shown in Table 1, as well as the adjusted R-square and the root mean squared error (RMSE). This indicates that the fitting is good.

With these experiments, the characteristics of the tactile sensor for velocity measurement can be obtained as shown in Table 2. It indicates that the tactile sensor has a good linearity along the *x*- and *y*- directions, which verifies the aforementioned model Equation (5) and confirms the feasibility of the proposed tactile sensor for velocity measurement, which is the basis of detecting stick-slip interaction.

### 4.2. Stick-Slip Detection with the Tactile Sensor

With the above setup, we have also carried out the test of stick-slip detection. The test in the *x*-direction is also only described here. Let the motion platform move up along *z*-axis to keep the tactile sensor contacting with the wafer, and then make the tactile sensor move with a constant velocity along the *x*-axis (*V*_const*-x*_) until the slip occurs on the contact surface, while recording simultaneously the outputs of Nano 17 and the output of the tactile sensor with respect to time. For all signals, the sample is set as 5 kHz in the NI PCIe-6323.

**Figure 11:**
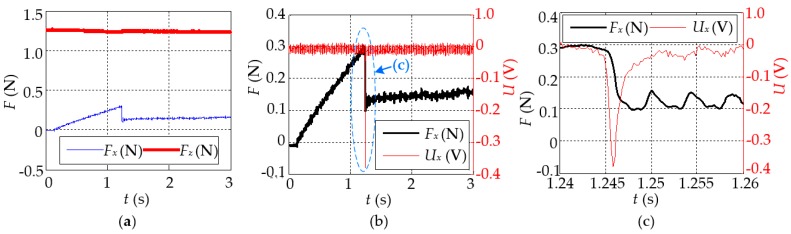
The result of stick-slip detection in the case that *V*_const*-x*_ = 0.6 mm/s. (**a**) The output of F/T sensor (Nano 17); (**b**) the output of tactile sensor in the *x*-direction *U_x_* and *F_x_* are shown in the same picture, and the part in the dashed circle is enlarged as shown in (**c**).

Figure 11 illustrates the experimental result in the case that the tactile sensor moves with a typical speed (here, *V*_const*-x*_ = 0.6 mm/s) under *F_z_* = 1.24 N. This indicates that the friction increases gradually with a certain slope from 0 in the beginning, up to the peak (*F_x_* = 0.30 N) around *t* = 1.243 s, then drops suddenly to a smaller value (*F_x_* ≈ 0.13 N), and subsequently remains this value for a long time (Figure 11a). The whole process is completely consistent with the aforementioned assumptions; that is, the contact surface has experienced successively “stick”, “stick-slip”, and “slip” states and the friction has changed obviously from the static friction *F_s_* = 0.30 N down to *F_c_* = 0.13 N. The measurement result of the tactile sensor (*U_x_*) is shown in Figure 11b, as well as the friction (*F_x_*) in the same figure. It illustrates the sensor output has an obvious change when the “stick-slip” appears. In order to display more details, the “stick-slip” part is enlarged as shown in Figure 11c, which demonstrates clearly that the tactile sensor can respond to the change of friction with about 1 ms in advance during the “stick-slip” state.

**Figure 12:**
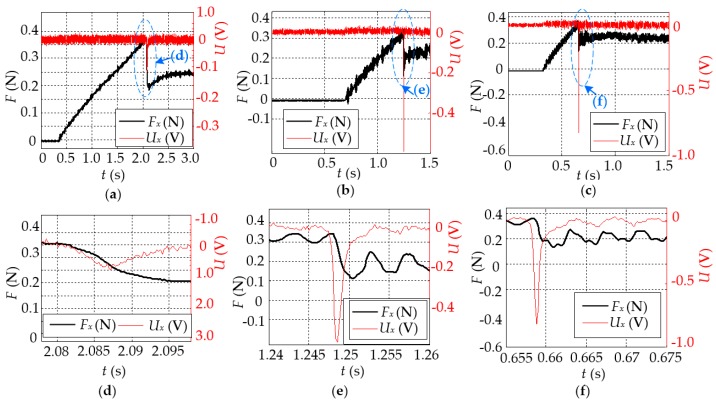
The results of stick-slip detection under different *V*_const*-x*_. (**a**) The result in the case that *V*_const*-x*_ = 0.3 mm/s, and the part in the dashed circle is enlarged as shown in (**d**); (**b**) the result in the case that *V*_const*-x*_ = 1.0 mm/s, and the part in the dashed circle is enlarged as shown in (**e**); and (**c**) the result in the case that *V*_const*-x*_ = 2.0 mm/s, and the part in the dashed circle is enlarged as shown in (**f**).

Figure 12a–c show the results in the cases that *V*_const*-x*_ = 0.3 mm/s, 1.0 mm/s, and 2.0 mm/s, respectively, and those for the corresponding “stick-slip” are enlarged as shown in Figure 12d–f, respectively. This indicates, for all cases, that the tactile sensor can respond to the change of friction with about 1 ms in advance during the “stick-slip” state as similar to that in Figure 11. All of them confirm that the output of the tactile sensor can be used directly to detect stick-slip on the contact surface; that is, *J*(*t*) = *U*(*t*) in Equation (11) is feasible for determining the stick-slip, which verifies the feasibility of the proposed method of stick-slip detection.

For detecting stick-slip, an appropriate threshold value (*J*_threshold_) for the judgment function *J*(*t*) is critical and needs to be determined. As *J*(*t*) = *U*(*t*), *J*_threshold_ can be obtained directly from *U*(*t*). Apparently, the maximum amplitude of *J*(*t*) during the “stick” and the “slip” is a reasonable value as a threshold, that is, |*J*_threshold_| = max{|*U*_stick_(*t*)|, |*U*_slip_(*t*)|}. From all the results in Figure 11 and Figure 12, we can obtain |*J*_threshold_| = 0.05 V, which is suitable for all of cases to detect stick-slip.

## 5. Discussion

**Figure 13:**
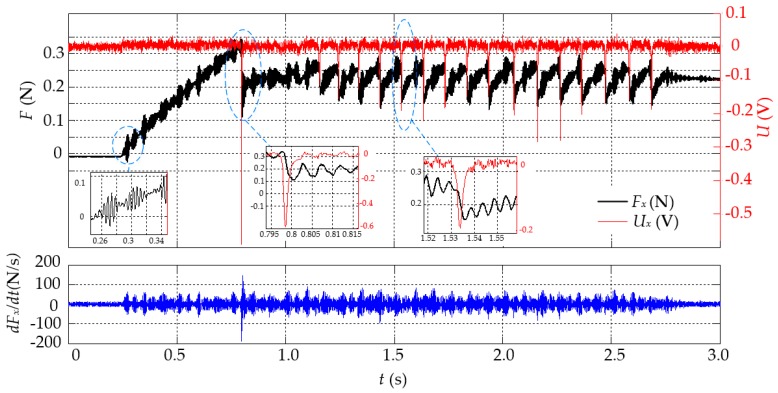
The result of stick-slip detection in the case that *V*_const*-x*_ = 1.0 mm/s in the period of 3.0 s.

Though the results in Figure 11 and Figure 12 show the tactile sensor is capable of detecting stick-slip, all of these cases are those with one “stick-slip”. Figure 13 shows the result of a typical case with continuous “stick-slip” states happening. It demonstrates that after the first “stick-slip” happening there are also several ones subsequently and the tactile sensor can response to the change of friction for each stick-slip in advance, which indicates that the tactile sensor is able to detect the continuous stick-slip interaction. The result also indicates that the judgment function *J*(*t*) = *U*(*t*) Equation (11) is feasible for determining the stick-slip and |*J*_threshold_| = 0.05 V is a reasonable value for stick-slip detection.

From Figure 13, it must be noted that *U_x_* varies with the “falling” of friction, but not just the first-order derivative change of friction (*dF_x_*/*dt*), which implies the relationship between *U_x_* and the change of friction is more complex. Actually, the friction phenomenon is inherently complex, due to many factors including the nonlinearity of materials (like elasto-plasticity) and contact nonlinearity [30]. This issue is beyond the scope of this paper. However, the amplitude of *U_x_* mostly increases with the amplitude of friction-“falling” during stick-slip, especially with recognizing the change direction, which is the outstanding feature of the proposed tactile sensor, compared with other tactile sensors for slip detection with other principles [5,11]. Furthermore, for a real specific application, *J*_threshold_ should be determined by abundant experimental data as the characteristics of friction for different contact pairs are various due to different materials.

Meanwhile, it must be noted that the experimental result also indicates that the micro-vibration of friction appears in the “stick” and “slip” states. For more details, two corresponding regions are enlarged as shown in Figure 13, where the results in the periods of *t* = 0.25–0.35 s and *t* = 1.52–1.56 s, respectively. It further indicates that the micro-vibration of friction can be considered as the micro stick-slip interaction in a local contact region during the “stick”, and the following big jump of friction as the macro stick-slip interaction in the whole contact region, which is consistent with the results in [16,18]; that is, the sliding appears in a local contact region and then suddenly extends to the whole contact region from “stick” to “slip”. This feature confirms that the tactile sensor is able to be used to detect the incipient slipping. Additionally, the results in Figure 11 and Figure 12 also show that the micro-vibration amplitude of friction increases along with the sensor moving with a bigger speed from “stick” to “slip” on the wafer surface. However, this issue is beyond the scope of this paper.

## 6. Conclusions

In this paper, we proposed a novel tactile sensor with electromagnetic induction for detecting the stick-slip interaction. The tactile sensor has a compact structure with a special design, inspired by a velocity sensor based on electromagnetic induction. For obtaining dynamic contact information, an equivalent cantilever-beam model of the tactile sensor was built to construct the relationship between the sensor output and the friction applied on the sensor. Furthermore, a method of detecting stick-slip interaction on the contact surface with the tactile sensor was proposed based on the change characteristics of friction. For a typical application, stick-slip interaction testing on a wafer transfer robot, a prototype was designed according to the requirements of wafer transfer, especially considering the spatial magnetic field distribution of the magnet and the size of the magnet and chip-inductor array. By using an experimental setup with a 3D micro-motion and six-axis F/T sensor, the sensor calibration was conducted, as well as the test of stick-slip detection with the tactile sensor. The results indicate that the tactile sensor has good linearity for velocity measurement and is capable of capturing the friction change during the stick-slip on the contact surface between a wafer sample and the sensor (wafer transfer surface), which verifies the principle of the tactile sensor and the feasibility of the method for stick-slip interaction testing on the wafer transfer surface.

Additionally, it must be noted that the prototype has a compact structure, light weight, and low cost, as well as a good dynamic performance. The output of the tactile sensor is directly used for slip sensing, simpler than those indirect measurement methods, such as estimating the friction coefficient or friction angle [4,11]. The experimental results also confirm that the tactile sensor is able to respond to the stick-slip with about 1 ms in advance under a sampling rate of 5 kHz, better than most tactile sensors for slip perception [15]. Nevertheless, the performance of tactile sensor relies on the properties of the elastomer, such as elasticity and durability, all of which affect the sensor linearity, sensitivity, and life cycle. However, there are many elastic materials with low cost and good properties, such as silicon rubber and polyurethane, providing more choices for the design of the tactile sensor. Meanwhile, the elastomer with the magnet can be made as a standard unit, easy to be replaced if there is a problem with the elastomer of tactile sensor.

In the future, we will focus on its application on a wafer transfer robot, including the control method with the tactile sensing feedback for wafer transfer, and the development of the end effector used in the vacuum is also another ongoing task by taking the wiring and sensor mounting into account. Moreover, the tactile sensor also has the potential to be used in other robot-environment interaction systems for detecting the contact state, especially in those with soft-rigid surfaces, since the contact between wafer and tactile sensor here is a typical one.

## Figures and Tables

**Table 1:** The result of polynomial fittings for the tactile sensor.

*i*-Direction	*K_Ci_* (mm/sV)	*b_i_* (mm/s)	Adj. R-Square	REMS
*x*-direction	−692.52	4.83 × 10^−4^	0.9978	9.236 × 10^−4^
*y*-direction	−665.78	−1.37 × 10^−4^	0.9928	8.473 × 10^−4^

**Table 2:** The characteristics of the tactile sensor for velocity measurement.

*i*-Direction	Repeatability	Sensitivity	Nonlinearity
*x*-direction	3.66%	1.44 × 10^−3^ V/(mm/s)	5.37%
*y*-direction	3.71%	1.50 × 10^−3^ V/(mm/s)	4.57%

## References

[1] Haddadin S., Albu-Schaffer A., Hirzinger G. (2009). Requirements for safe robots: Measurements, analysis and new insights. Int. J. Robot. Res..

[2] Yoshikawa T. (2010). Multifingered robot hands: Control for grasping and manipulation. Annu. Rev. Control.

[3] Chen C., Chen J., Huang C., Lu J., Lin P. (2015). Sensor data fusion for body state estimation in a bipedal robot and its feedback control application for stable walking. Sensors.

[4] Dahiya R.S., Metta G., Valle M., Sandini G. (2010). Tactile sensing-from humans to humanoids. IEEE Trans. Robot..

[5] Girao P.S., Ramos P.M., Postolache O., Pereira J.M. (2013). Tactile sensors for robotic applications. Measurement.

[6] Yousef H., Boukallel M., Althoefer K. (2011). Tactile sensing for dexterous in-hand manipulation in robotics—A review. Sens. Actuat. A Phys..

[7] Palli G., Melchiorri C., Vassura G., Scarcia U., Moriello L., Berselli G., Cavallo A., Maria G.D., Natale C., Pirozzi S. (2014). The dexmart hand: Mechatronic design and experimental evaluation of synergy-based control for human-like grasping. Int. J. Robot. Res..

[8] Puangmali P., Althoefer K., Seneviratne L.D., Murphy D., Dasgupta P. (2008). State-of-the-art in force and tactile sensing for minimally invasive surgery. IEEE Sens. J..

[9] Abushagur A., Arsad N., Reaz M., Bakar A. (2014). Advances in bio-tactile sensors for minimally invasive surgery using the fibre bragg grating force sensor technique: A survey. Sensors.

[10] Liu T., Inoue Y., Shibata K. (2009). A small and low-cost 3-D tactile sensor for a wearable force plate. IEEE Sens. J..

[11] Francomano M.T., Accoto D., Guglielmelli E. (2013). Artificial sense of slip—A review. IEEE Sens. J..

[12] Cavallo A., Maria G.D., Natale C., Pirozzi S. (2014). Slipping detection and avoidance based on Kalman filter. Mechatronics.

[13] Cotton D.J., Chappell P.H., Cranny A., White N.M., Beeby S.P. (2007). A novel thick-film piezoelectric slip sensor for a prosthetic hand. IEEE Sens. J..

[14] Fernando V., Maria J.B., Julián C., Rafael N., Jose A., Javier S. (2011). A large area tactile sensor patch based on commercial force sensors. Sensors.

[15] Fernandez R., Payo I., Vazquez A., Becedas J. (2014). Micro-vibration-based slip detection in tactile force sensors. Sensors.

[16] Ho V., Dao D., Sugiyama S., Hirai S. (2011). Development and analysis of a sliding tactile soft fingertip embedded with a microforce/moment sensor. IEEE Trans. Robot..

[17] Zhang Y., Yi J. (2014). Static tire/road stick-slip interactions: Analysis and experiments. IEEE/ASME Trans. Mechatron..

[18] Trkov M., Han H., Yi J., Liu Y. Stick-slip interactions of the soft-solid contact: An intergrated LuGre/beam network model approach. Proceedings of the ASME 2015 Dynamic Systems and Control Conference.

[19] Martino M., Danisi A., Losito R., Masi A., Spiezia G. (2010). Design of a linear variable differential transformer with high rejection to external interfering magnetic field. IEEE Trans. Magn..

[20] Kim Y., Kim S., Park K. (2009). Magnetic force driven six degree-of-freedom active vibration isolation system using a phase compensated velocity sensor. Rev. Sci. Instrum..

[21] Wattanasarn S., Noda K., Matsumoto K., Shimoyama I. 3D flexible tactile sensor using electromagnetic induction coils. Proceedings of the IEEE 25th International Conference on Micro Electro Mechanical Systems.

[22] Han H., Liu Y., Liu T., Inoue Y., Shibata K. A novel velocity sensor based on electromagnetic induction. Proceedings of the 2011 IEEE Sensors.

[23] Cheng H.T., Chen H.P., Mooring B.W. (2014). Accuracy analysis of dynamic-wafer-handling robotic system in semiconductor manufacturing. IEEE Trans. Ind. Electron..

[24] Liu Y., Xu M., Cao Y. (2012). Research, design and experiment of end effector for wafer transfer robot. Ind. Robot.

[25] Liu Y., Cao Y., Sun L., Zheng X. (2010). Accurate and steady control on trajectory tracking for the wafer transfer robot. Ind. Robot.

[26] Liu Y., Wu J., Li K., Han H., Liu T. A detection method of stick-slip state on wafer transfer surface and its sensor design. Proceedings of the IEEE International Conference on Mechatronics and Automation (ICMA).

[27] Tumanski S. (2007). Induction coil sensors—A review. Meas. Sci. Technol..

[28] Beer F.P., Johnston E.R., DeWolf J.T., Mazurek D.F. (2012). Mechanics of Materials.

[29] Furlani E.P. (2001). Permanent Magnet and Electromechanical Devices.

[30] Johanastrom K., Canudas-de-Wit C. (2008). Revisiting the lugre friction model. IEEE Control Syst..

